# Combination of Pericapsular Nerve Group (PENG) and Sacral Erector Spinae Plane (S-ESP) Blocks for Hip Fracture Pain and Surgery: A Case Series

**DOI:** 10.7759/cureus.53815

**Published:** 2024-02-07

**Authors:** Francesco Marrone, Pierfrancesco Fusco, Serkan Tulgar, Saverio Paventi, Marco Tomei, Fabio Fabbri, Michele Iacovazzi, Carmine Pullano

**Affiliations:** 1 Anesthesiology, Santo Spirito Hospital, Rome, ITA; 2 Anesthesiology and Intensive Care Unit, San Filippo e Nicola Hospital, Avezzano, ITA; 3 Anesthesiology, Samsun University Faculty of Medicine, Samsun, TUR; 4 Anesthesiology and Critical Care, Santo Spirito Hospital, Rome, ITA; 5 Anesthesiology and Critical Care, Azienda Sanitaria Locale - Roma 1 (ASL Roma 1), Rome, ITA; 6 Anesthesiology and Critical Care, Azienda Sanitaria Locale - Bari (ASL Bari) Ospedale Della Murgia "Fabio Perinei", Bari, ITA; 7 Anesthesiology, Villa Pia Clinic, Rome, ITA

**Keywords:** regional anesthesia, local anesthetic adjuvants, geriatric hip fracture, pericapsular nerve group block (peng), sacral erector spinae plane block

## Abstract

A hip fracture is a serious injury with life-threatening complications, and its risk rises with increasing age. A hip fracture can be a very painful condition, and prompt surgical treatment is recommended to reduce pain and complications. Pain management is considered integral to the management of a broken hip. The choice between general and regional anesthesia in hip fracture surgery continues to be a topic of debate because risks are potentially associated with both approaches. Nerve blockades have proven to be effective in reducing acute pain after a hip fracture and in the perioperative period. For this reason, many regional techniques have been introduced, such as the lumbar plexus block, fascia iliac block, femoral nerve block, and recently, the pericapsular nerve group (PENG) block. Hip joint innervation is complex, not limited to the lumbar plexus but also depending on the sciatic nerve and branches of the sacral plexus (superior and inferior gluteal nerves and an articular branch from the quadratus femoris nerve). We hypothesized that a combination of two emerging regional anesthesia techniques, such as the PENG block and sacral erector spinae plane (S-ESP) block, could represent a good option to obtain pain control of the whole hip joint without opioid administration intraoperatively and postoperatively. Here, we report the cases of three frail patients with significant comorbidities who underwent hip fracture surgery (two cases of intramedullary nailing and one hemiarthroplasty), in which we preoperatively performed PENG and S-ESP blocks. We registered optimal intraoperative and postoperative pain control up to 48 hours after surgery without complications and without opioid administration, allowing the surgery to be performed with intravenous sedation or laryngeal mask general anesthesia. The surgeries were uneventful, and no complications were reported. This approach warrants further investigation in hip fracture surgery.

## Introduction

A broken hip (hip fracture) in elderly and frail patients is a serious injury with complications that can be life-threatening and needs immediate treatment in the hospital. Surgery is the preferred form of treatment within the first 24-48 hours to reduce complications and improve outcomes [1]. Depending on the age and the type of break, surgical options range from fixing broken bones back into place using screws, nails, rods, or plates to replacing some of the damaged hip joints with an artificial part (hemiarthroplasty) or replacing all of the damaged hip joints with an artificial joint (hip replacement).

Pain management is considered integral to the management of a broken hip. Intraoperative pain management has traditionally been achieved with systemic analgesia in association with general anesthesia. Despite this, neuraxial anesthesia is gaining popularity as a substitute for general anesthesia for its short-term benefits, such as reduction in pulmonary complications [2], venous thromboembolism and the need for blood transfusions [3], less postoperative nausea (no opioid use) [4], and potentially less risk of postoperative delirium due to minimal or no intraoperative sedation [5, 6]. The choice between general and regional anesthesia continues to be a topic of debate [7] because risks are potentially associated with both approaches. Long-term outcomes (recovery of ambulation, survival, and return to pre-trauma residence at 60 days) showed to be similar with spinal versus general anesthesia in patients who were 50 years of age and older [8]. To date, there is a scarcity of literature addressing real-world determinants of anesthesia-type decisions in hip fracture surgery. Intraoperative and postoperative pain management is achieved by an expanded range of interventions, including systemic analgesia, regional nerve blocks, and physical therapy. Nevertheless, the only intervention that was found to be effective at reducing acute pain in hip fractures proved to be peripheral nerve blocks [9]. Hip joint innervation is quite complex, and understanding the nerves targeted by different approaches is crucial to choosing which block to perform. There are several regional techniques employed for their opioid-sparing analgesic potential (fascia iliaca block, femoral nerve block, lumbar plexus block, and pericapsular nerve group (PENG) block), but no consensus exists about which block to perform preoperatively and in a specific surgical setting (surgery to replace all or a part of the hip joint, surgery to fix a fracture with a plate and/or screws, or surgery to fix a fracture with a rod inside the thigh bone) [10]. These regional techniques are unsuitable for providing surgical anesthesia and comprehensive analgesia for the entire hip joint, as they do not fully cover its complex innervation.

Here, we report a non-conventional perioperative pain management strategy in three frail patients with significant comorbidities who underwent hip fracture surgery (two cases of intramedullary nailing and one hemiarthroplasty). To achieve optimal intraoperative and postoperative pain control as well as the most comprehensive coverage of hip joint innervation without neuraxial techniques or opioid administration, we postulated that two regional anesthesia techniques, such as PENG, and sacral erector spinae plane (S-ESP) blocks could represent the most effective and intriguing choice.

## Case presentation

The patients' written consent was obtained for the preoperative placement of a combination of PENG block and S-ESP block to provide intraoperative and postoperative pain control. This case report was written in accordance with the CAse REport (CARE) guidelines for case reports [11]. Patients' characteristics and clinical data are summarized in Table 1.

**Table 1:** Patients' characteristics and clinical data BMI: body mass index; ASA: American Society of Anesthesiologists; NRS: numeric rating scale; PENG: pericapsular nerve group; S-ESP: sacral erector spinae plane; dexa: dexamethasone; dexm: dexmedetomidine; RA: regional anesthesia; LMA: laryngeal mask; GA: general anesthesia

	Patient one	Patient two		Patient three
Age (in years)	84	87		92
Sex	Female	Female		Female
BMI (kg/m^2^)	18	26		22
Weight (kg)	50	75		55
ASA classification	III	III		III
NRS score: PACU discharge	2	3		3
NRS score: 18 hours after surgery	4	4		4
NRS score: 36 hours after surgery	3	3		3
Dosing for PENG block	0.2% ropivacaine 20 mL + dexm 15 mcg + dexa 3 mg	0.2% ropivacaine 20 mL		0.2% ropivacaine 20 mL
Dosing for S-ESP block	0.2% ropivacaine 35 mL + dexm 15 mcg + dexa 3 mg	0.2% ropivacaine 30 mL + dexm 25 mcg + dexa 4 mg		0.25% ropivacaine 30 mL + dexm 20 mcg + dexa 4 mg
Block coverage	L1-S3	L1-S3		L1-S3
Type of surgery	Nailing		Nailing	Hemiarthroplasty
Surgery duration (min)	71		64	86
Anesthesia type	RA + sedation		RA + sedation	RA + LMA GA

Case one

An 84-year-old female patient, American Society of Anesthesiologists (ASA) class III, 50 kg in weight and 166 cm in height, with a body mass index (BMI) of 18 kg/m^2^, was proposed for hip fracture surgery (gamma-nail synthesis). The patient suffered from emphysema, hypertension, non-insulin-dependent diabetes, atrial fibrillation, and anticoagulation with antivitamin K (AVK), and she was scheduled for surgery the day after her arrival.

Under standard monitoring, intravenous midazolam (1 mg) was given for sedation. First, with the patient in a supine position, we provided an aseptic US-guided (Edge II, FUJIFILM-Sonosite^TM^, Bothwell, WA) PENG block injecting 20 ml of 0.2% ropivacaine with dexmedetomidine 15 mcg + dexamethasone 3 mg. Twenty minutes after blockade placement, when pain on movement was reduced, the patient was placed on the non-fractured limb, and in an aseptic condition, we administered at sacral 1 (S1) intermediate sacral crest, 35 ml of 0.2% ropivacaine with dexmedetomidine 15 mcg + dexamethasone 3 mg. Forty minutes after the procedure, we registered full sensory blockade in the lumbar and sacral nerves (L1-S3) bilaterally without the motor block.

The patient was introduced into the operating theatre and positioned on a traction bed without pain. We started intravenous sedation by providing 3 mg of midazolam + ketamine (total 100 mg: 20 mg repeated five times) to the spontaneously breathing patient (oxygen nasal cannula 4 l.min^-1^). Surgeons did not use local anesthetics for infiltration, and the absence of a motor block was not challenging. The surgery lasted 71 minutes without bradycardia or arterial hypotension. No opioids were provided in the intraoperative period. The patient was transferred to the post-anesthesia care unit (PACU) with an Aldrete score of nine and a numeric rating scale (NRS) score of two. In the ward, we registered an NRS score of four after 18 hours from blockade placement with the first analgesic request (1 g of paracetamol). The postoperative pain regimen included only paracetamol which was prescribed three times a day.

Case two

An 87-year-old female patient was proposed for hip fracture surgery (gamma-nail synthesis). She was 170 cm in height and weighed 75 kg with a BMI of 26 kg/m^2^. Her comorbidities included arterial hypertension and chronic heart failure, and she was classified as ASA class III.

Under standard monitoring, intravenous midazolam (1 mg) was given for sedation. First, with the patient in a supine position, we provided an aseptic US-guided PENG block by injecting 20 ml of 0.2% ropivacaine without additives. Ten minutes after blockade placement, when dynamic pain was reduced, the patient was placed on the non-fractured limb, and in an aseptic condition, we administered at S1 intermediate sacral crest, 30 ml of 0.2% ropivacaine with dexmedetomidine 25 mcg + dexamethasone 4 mg. Forty minutes after the procedure, we registered sensory blockade in the lumbar and sacral nerves (L1-S3) bilaterally without the motor block.

The patient was introduced into the operating theatre and positioned on a traction bed without pain. Sedation was initially provided by intravenous midazolam (3 mg) + 50 mg of propofol and then via intravenous dexmedetomidine infusion 0.5 mcg.Kg^-1^.h^-1^, with a spontaneously breathing patient (oxygen nasal cannula 4 l.min^-1^). Surgeons did not use local anesthetics for infiltration, and the absence of motor blocks was not challenging. The surgery lasted 64 minutes without bradycardia or arterial hypotension. No opioids were provided in the intraoperative period. The patient was transferred to the PACU with an Aldrete score of 10 and an NRS score of three. In the ward, we registered an NRS score of four after 18 hours from blockade placement with the first analgesic request (1 g of paracetamol). The postoperative pain regimen included only paracetamol prescribed three times a day.

Case three

A 92-year-old patient, ASA class III, was scheduled for hip fracture surgery (hemiarthroplasty). She was 160 cm in height and weighed 55 kg with a BMI of 22 kg/m^2^. Her medicines included apixaban for atrial fibrillation, and she was proposed for surgery the day after her arrival.

Under standard monitoring, intravenous midazolam (1 mg) was given for sedation. First, with the patient in a supine position, we provided an aseptic US-guided PENG block by injecting 20 ml of 0.25% ropivacaine without additives. After that, from blockade placement, when dynamic pain was reduced, the patient was placed on the non-fractured limb, and in an aseptic condition, we administered at S1 intermediate sacral crest, 30 mL of 0.25% ropivacaine with dexmedetomidine 20 mcg + dexamethasone 4 mg.

The patient was introduced in the operating room, and general anesthesia was provided via desflurane minimum alveolar concentration (MAC) 0.5 and a laryngeal mask with a fraction of inspired oxygen (FiO_2_) 0.4. General anesthesia was administered to ensure the surgeon had a higher level of muscle relaxation for performing hemiarthroplasty. Surgeons did not use local anesthetics for infiltration. The surgery lasted 86 minutes without bradycardia or arterial hypotension. No opioids were provided in the intraoperative period. After emergence from general anesthesia, the patient was transferred to the PACU with an Aldrete score of 10 and an NRS score of three. In the ward, we registered an NRS score of four after 18 hours from the placement of the blocks with the first analgesic request (1 g of paracetamol). The postoperative pain regimen included only paracetamol which was prescribed three times a day.

## Discussion

Hip fracture surgery in the elderly is challenging because of multiple comorbidities. When formulating an anesthetic plan, we had to consider the challenges with airway management, respiratory disease, cardiovascular compromise, the use of medications such as anticoagulant or platelet antiaggregant drugs, and intraoperative and postoperative analgesic requirements. We also considered the possibility of local anesthetic systemic toxicity if multiple regional techniques were used in combination. After a risk-benefit discussion with the patients, we planned to perform a combination of two blocks, the S-ESP block and the PENG block, as a primary analgesic technique together with intravenous sedation for the intramedullary nailing or laryngeal mask general anesthesia in the case of hemiarthroplasty, for optimal intraoperative and perioperative pain control without opioid administration and avoiding neuraxial techniques.

**Figure 1:**
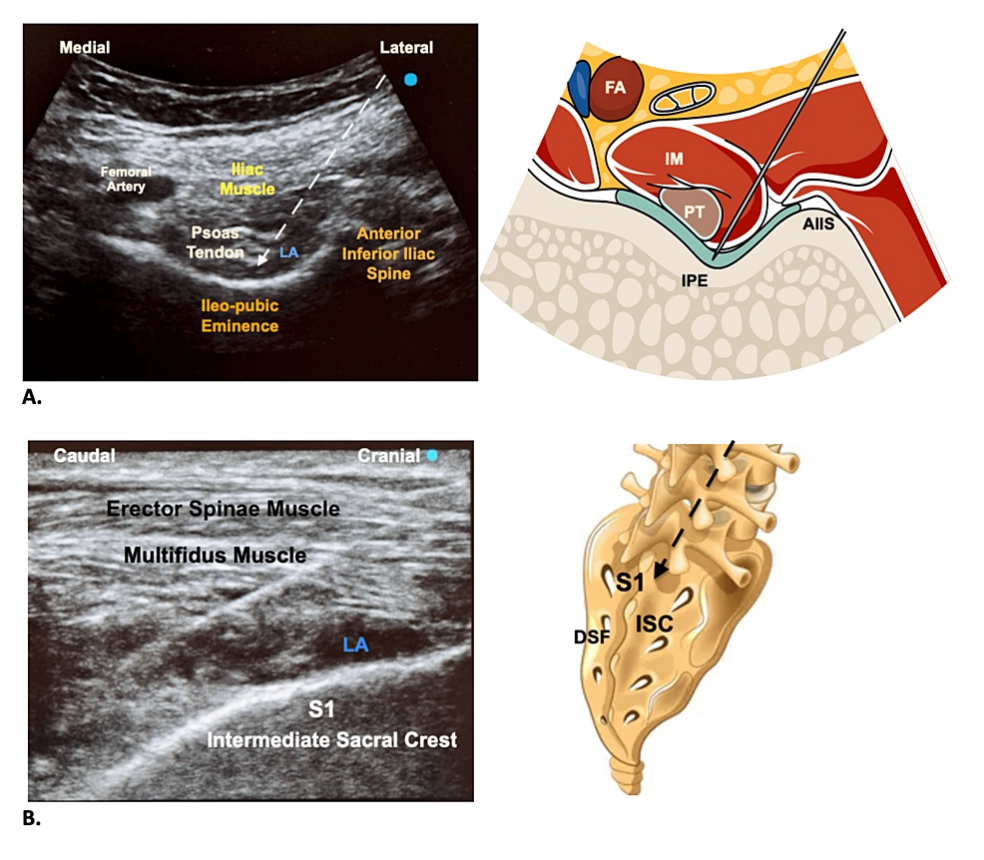
Pericapsular nerve group (PENG) and sacral erector spinae plane (S-ESP) blocks A: Sonoanatomy and graphical representation of the PENG block; B: Sonoanatomy of the S-ESP block and graphical representation of the sacrum. The dotted arrow shows a craniocaudal puncture. LA: local anesthetic; FA: femoral artery; IM: iliac muscle; PT: psoas tendon; IPE: ileopubic eminence; AIIS: anterior inferior iliac spine; DSF: dorsal sacral foramina; S1: sacral 1; ISC: intermediate sacral crest Scan images are from patient one's records. The graphical images are the original work of the authors.

The PENG block was introduced in hip fracture surgery in 2018 [12]. It showed the potential of motor-sparing analgesia by blocking the (anterior) articular branches of the hip joint [13], but it can’t represent an anesthesia option for hip surgery, showing some benefit to postoperative analgesia [14]. The S-ESP block was introduced in 2019 to achieve blockage of posterior sacral nerves in pilonidal cyst surgery [15], and since then its application in perineal, anorectal, hip, and lower limb surgeries has been reported [16-19]. The S-ESP block consists of injecting local anesthetic under the multifidus muscle plane, obtaining sacral nerve root block, and potentially blocking the lumbar plexus (L1-L4) via the cephalad and epidural spread of local anesthetic (Figure 1) [20].

The innervation of the hip joint is complex because it is not limited to the lumbar plexus alone, as the lateral cutaneous branch of the ilioinguinal nerve, the lateral cutaneous branch of the subcostal nerve, the superior and inferior gluteal nerves, the nervus quadratus femoris, and perhaps most overlooked and indeed crucially, the superior cluneal nerve, all play active roles [21-23]. Due to this complex hip innervation, we hypothesized to target the posterior aspect and posterolateral cutaneous innervation of the hip joint with S-ESP, while with PENG block, we aimed for the anterolateral aspect of the hip capsule (sometimes achieving cutaneous blockade with a high volume). A suprainguinal fascia Iliaca approach could represent an alternative option for the PENG block as it can have significant spread to the three target nerves of the lumbar plexus, but the suggested volume to reach the femoral, obturator, and lateral femoral cutaneous nerves was 40 mL [24], with the need for larger volumes and consequently higher doses of the local anesthetic. We found the combination of S-ESP and PENG blocks to be highly successful in our cases, overcoming the limitations of neuraxial techniques and the risks of general anesthesia and providing long-lasting postoperative analgesia (up to 48 hours). Nevertheless, we registered full sensory blockade ranging from L1 to S3 dermatomes. Hip surgery should include T12; here, the superior cluneal nerve and the lateral cutaneous branches of the subcostal-iliohypogastric nerves have escaped. This circumstance made it necessary to add an intravenous regimen of analgosedation to overcome pain in the skin incision. We are not aware if the anesthesia quality would increase if we performed a lumbar ESP block from L1.

A combination of blocks certainly warrants augmented risks of local anesthetic systemic toxicity (LAST), especially in elderly and frail patients. Nevertheless, combinations of blocks should be tried by adjusting the injectate volumes and local anesthetic concentrations. As described in the literature, the perineural dexamethasone-dexmedetomidine combination could be a "formula," as it has proven to be effective in enhancing peripheral nerve blocks [25, 26] and analgesia duration in the fascial blocks [27, 28], making it possible to use larger volumes of a diluted local anesthetic [19]. Further studies are warranted to highlight the full potential and risks of the perineural dexamethasone-dexmedetomidine combinations.

The advantages of the PENG and S-ESP block combination include simplicity of execution, the absence of contraindications related to some medications, such as platelet antiaggregants or anticoagulants, and the absence of motor weakness while producing analgesia. Potential disadvantages are the placement of two blocks, the slow onset time, and the unpredictability of dermatomeric coverage in the S-ESP block. The T12 coverage exclusion made mandatory the use of an intravenous analgosedation regime (intravenous midazolam/ketamine in the first case due to pain in the skin incision and intravenous midazolam/propofol/dexmedetomidine in the second, as the pain was felt at the cephalic end of the surgical incision). Another disadvantage could be represented by the absence of a motor block, which could represent a challenge for the surgeons, especially in the case of hemiarthroplasty. For this kind of surgery, we preferred laryngeal mask general anesthesia.

## Conclusions

In our cases, the combination of PENG and S-ESP blocks proved to be a good analgesia option for hip fracture surgery in frail patients, overcoming the limitations of neuraxial techniques and the risks of general anesthesia and providing long-lasting postoperative analgesia. This approach warrants further investigation in hip fracture surgery and potentially in elective hip joint replacement, aiming to improve the postoperative recovery trajectories of orthogeriatric patients. Anatomical studies and further investigation are needed to better explain the potential of the S-ESP block.
